# Associations of risk and time preferences with Japanese workers' physical activity and sedentary behavior

**DOI:** 10.1016/j.pmedr.2026.103491

**Published:** 2026-05-05

**Authors:** Yoshino Hosokawa, Kaori Ishii, Ai Shibata, Koichiro Oka

**Affiliations:** a Faculty of Health and Sports Sciences, Toyo University, Kita, Japan; b Waseda Institute for Sport Sciences, Waseda University, Tokorozawa, Japan; c Faculty of Sport Sciences, Waseda University, Tokorozawa, Japan; d Institute of Health and Sport Sciences, University of Tsukuba, Tsukuba, Japan

**Keywords:** Time preference, Risk preference, Behavioral economic, Physical activity, Sedentary behavior, Japanese workers

## Abstract

**Objective:**

Although individual risk and time preferences appear to be associated with (un)healthy behaviors, limited evidence exists regarding whether domain-specific physical activity and sedentary behaviors vary according to risk and time preferences. This study investigated the association of risk and time preference with domain-specific physical activity and total sedentary time among Japanese working adults.

**Methods:**

This study recruited 2400 Japanese workers via an internet survey conducted in March 2022 and assessed their risk preferences, time preferences, domain-specific physical activity times, total sedentary time, and sociodemographic characteristics. Data were analyzed using a multiple regression analysis.

**Results:**

Multiple regression analysis indicated that a risk-averse disposition in risk preference was significantly associated with lower total sedentary time. No statistically significant relationship was observed between risk preference and any physical activity measure. A higher time preference was significantly associated with higher moderate-to-vigorous physical activity during work and with lower total sedentary time.

**Conclusions:**

Individual risk preferences were not clearly related to domain-specific physical activity. In contrast, higher time preference was modestly associated with physical activity in work-related contexts. Given that both risk and time preferences were linked to total sedentary time, further research is needed to examine sedentary behavior across domains.

## 1 Introduction

Numerous studies have demonstrated that physical inactivity and excessive sedentary behavior are risk factors for mortality associated with non-communicable diseases, such as cardiovascular disease, type II diabetes, and certain types of cancers, thus highlighting the urgent need to promote preventive measures worldwide (Biswas et al., 2015). However, changing daily behavioral patterns is not easily achievable because sedentary lifestyles remain prevalent. Individuals who are physically inactive may lack sufficient motivation or preference to invest time and effort in physical activity for future health benefits (Nicholls and Watson, 2024). To develop effective strategies for encouraging a physically active lifestyle, it is vital to gain a detailed understanding of the factors related to individual decision-making regarding physical activity and sedentary behavior.

Several studies have applied concepts from behavioral economics, such as risk and time preferences, to explain health behaviors. For example, risk aversion, defined as a preference for certainty over risk even when expected values are similar, was reported to be positively associated with undergoing physical examinations (Herberholz, 2020) but negatively associated with unhealthy behaviors such as smoking and heavy alcohol consumption (Anderson and Mellor, 2008). High time preference, defined as an inclination to favor smaller immediate rewards over larger delayed rewards, is positively associated with smoking and unhealthy diet (Barlow et al., 2016; Kang and Ikeda, 2016). Previous research on the relationship between physical activity and risk preference is limited, and a few studies in the literature have found no statistically significant association between the two (van der Pol et al., 2017; Nicholls and Watson, 2024). By contrast, time preference has been reported to be significantly associated with physical activity, suggesting that lower time preference may be associated with higher physical activity levels across different domains (Kosteas, 2015; Shuval et al., 2017; Hunter et al., 2018). Given the interdependence between risk and time preferences (Andersen et al., 2008), it is important not to focus solely on one or the other but to consider their mutual influence when examining health behaviors such as physical activity (Nicholls and Watson, 2024).

Although previous studies on physical activity have examined a mixture of activities undertaken for different purposes, limited evidence exists regarding whether risk and time preferences vary according to domain-specific types of physical activity. Furthermore, little is known about their association with sedentary behaviors. Physical activity in discretionary contexts, such as leisure, may be influenced by individual decision-making (Kosteas, 2015), whereas activity in structured contexts, such as work, is typically shaped by external constraints (van Kasteren et al., 2020). Understanding these relationships across specific domains of physical activity is particularly important as motivations and decision-making processes influencing engagement may differ across occupational, transport, and leisure contexts. Examining such domain-specific patterns could provide valuable insights for the development of more effective and context-tailored interventions to promote physical activity. This study has investigated the association of risk and time preference with domain-specific physical activity and total sedentary time among Japanese working adults, a group particularly concerned with physical inactivity and excessive sedentary behavior.

## 2 Methods

### 2.1 Data source and participants

This study was a web-based cross-sectional survey conducted in March 2022. The survey was conducted via MyVoice Communications, Inc., an online research company with approximately 1.1 million registered participants. The company randomly sent email invitations containing a link to the questionnaire to 8860 workers aged 20–59 years across Japan. Participants were stratified by sex and age (20s, 30s, 40s, and 50s). The target sample size was 2400, and the survey was closed once this number was reached. All of the participants provided web-based informed consent before completing the questionnaires. As an incentive, the participants received 123 points redeemable at partner facilities. This study was approved by the Institutional Ethics Committee of Waseda University (2021–417).

### 2.2 Measures

#### 2.2.1 Risk and time preferences

Risk preference was assessed using a monetary lottery task, a widely used approach in behavioral economics that has been shown to be associated with general measures of risk preference (Dohmen et al., 2011). Participants were asked whether they would purchase a “speed lottery” ticket at a series of increasing prices (e.g.,10 Japanese yen, 2000 yen, 4000 yen, and up to 50,000 yen; nine price levels) under a scenario in which there was a 50% chance of winning 100,000 yen (approximately 670 US dollars) and a 50% chance of winning nothing. For each price, the participants chose Option A (“buy”) or Option B (“not buy”). Based on the highest price at which the participant was willing to purchase a ticket, an ordinal risk preference variable was constructed following Morimoto (2018), ranging from one (willingness to purchase at all price levels; highest risk taking) to nine (unwilling to purchase at any price level; highest risk aversion).

The time preference was assessed using the Multiple Price List (MPL) method (Coller and Williams, 1999). Participants were presented with nine paired choices between Option A (receiving a smaller amount of money sooner, 90 days from today) and Option B (receiving a larger amount later, 97 days from today) with the amount in Option B increasing across the nine choices, making Option B more attractive over nine successive comparisons. The participants selected their preferred options for each pair. Following Morimoto (2018), an ordinal time preference variable was constructed based on the point at which the participants switched from Option A to Option B with lower scores indicating greater patience (lower time preference) and higher scores indicating greater impatience (higher time preference). The participants who consistently selected Option B were assigned a score of whereas those who consistently selected Option A were assigned a score of 10. Those who switched multiple times or those who reversed from Option B to Option A excluded as invalid responses.

#### 2.2.2 Domain-specific physical activity and sedentary behavior

Domain-specific physical activity and total sedentary time were assessed using the Global Physical Activity Questionnaire (GPAQ) (Bull et al., 2009). The procedures for cleaning and scoring the GPAQ data have been comprehensively described elsewhere (World Health Organization, 2012). In summary, the participants self-reported the number of days per week and minutes per day engaged in moderate- and vigorous-intensity physical activity in the work and leisure domains, moderate-intensity physical activity in the transport domain over a typical week, and total minutes spent sedentary per day. This demonstrates acceptable reliability and validity among Japanese adults (Bull et al., 2009). The average weekly minutes of moderate-to-vigorous physical activity (MVPA) were calculated for each of the three domains. Total MVPA (minutes/week) was calculated as the sum of the three domains. Based on the GPAQ Analysis Guide (World Health Organization, 2012), implausible values for physical activity and sedentary time were treated as missing values.

#### 2.2.3 Sociodemographic characteristics

Participants reported their age, sex, educational attainment, marital status, and gross annual household income, occupational style, which were included as covariates.

### 2.3 Data analyses

Of the 2400 participants in this study, 1920 responses were included in the analysis, after excluding those with incomplete or inconsistent data across all analyzed items (Supplementary Fig. 1). Descriptive statistics, including frequencies and measures of central tendency and variability (i.e., means and standard deviations), were calculated for physical activity and sedentary time measures, as well as sociodemographic variables. A series of multiple regression analyses was used to examine the associations of risk and time preferences with physical activity and sedentary time measures. All models were adjusted for covariates. When the independent variables were physical activity measures, and total sedentary time was included as a covariate, or vice versa. Model assumptions were assessed by examining residual diagnostics, including normality, homoscedasticity, and influential outliers, using graphical methods. Statistical analyses were performed using IBM SPSS Statistics 29.0.

## 3 Results

Table 1 shows the characteristics of the study participants. The 1920 eligible participants exhibited an average of 457.5 ± 704.4 min/week of total MVPA time, with an average total sedentary time of 464.4 ± 250.6 min/day.

**Table 1:** Characteristics of participants in a web-based survey of Japanese workers aged 20–59 years, March 2022.

Variable	n or mean	% or SD
Age	41.4	10.5
Sex		
Men	936	48.8
Women	984	51.2
Educational attainment		
≤12 years	372	19.4
≥13 years	1548	80.6
Marital status		
Unmarried	1009	52.6
Married	911	47.4
Gross annual household income		
<5,000,000 Japanese yen [33,700 US dollars]	812	42.3
<10,000,000 Japanese yen [67,300 US dollars]	845	44.0
≥10,000,000 Japanese yen	263	13.7
Occupational style		
Sitting workers (sitting/desk-based)	1249	65.1
Non-sitting workers (standing/walking/physical labor)	671	34.9
Domain-specific physical activity and sedentary behavior		
Work-related moderate-to-vigorous physical activity time (minutes/week)	207.0	567.9
Transport-related physical activity time (minutes/week)	165.6	266.2
Leisure-related moderate-to-vigorous physical activity time (minutes/week)	84.9	174.7
Total moderate-to-vigorous physical activity time (minutes/week)	457.5	704.4
Total sedentary time (minutes/day)	464.4	250.6

SD, Standard deviation.

Table 2 presents the outcomes of the multiple regression analysis adjusted for covariates. Risk-averse disposition in risk preference was significantly associated with lower total sedentary time (β = −0.09, 95% CI: −14.41, −5.04). No statistically significant relationship was observed between risk preference and any physical activity measure. A higher time preference was significantly associated with higher MVPA during work (β = 0.06, 95% CI: 2.98, 18.56) and lower total sedentary time (β = −0.05, 95% CI: −7.80, −1.22).

**Table 2:** Associations of risk and time preferences with physical activity and sedentary behavior in a web-based survey of Japanese workers aged 20–59 years, March 2022.

	Risk preferences			Time preferences		
	B	β	95%CI	B	β	95%CI
Domain-specific physical activity and sedentary behavior						
Work-related moderate-to-vigorous physical activity time (minutes/week)	1.92	0.01	−9.22, 13.05	10.77	0.06	2.98, 18.56
R^2^ = 0.18, *p* < 0.01						
Transport-related physical activity time (minutes/week)	5.80	0.05	0.07, 11.53	−2.13	−0.02	−6.14, 1.87
R^2^ = 0.01, *p* = 0.01						
Leisure-related moderate-to-vigorous physical activity time (minutes/week)	−2.40	−0.03	−6.11, 1.31	−1.87	−0.03	−4.47, 0.72
R^2^ = 0.038, p < 0.01						
Total moderate-to-vigorous physical activity time (minutes/week)	5.32	0.02	−8.76, 19.40	6.77	0.030	−3.08, 16.61
R^2^ = 0.15, p < 0.01						
Total sedentary time (minutes/day)	−9.72	−0.09	−14.41, −5.04	−4.51	−0.05	−7.80, −1.22
R^2^ = 0.25, p < 0.01						

B, Unstandardized regression coefficient; β, Standardized regression coefficient; 95% CI, 95% confidence interval; R^2^, coefficient of determination.

Covariates: age, sex, educational attainment, marital status, gross annual household income, and occupational style. The independent variables were physical activity domains, and total sedentary time was included as a covariate (or vice versa).

*P*-values for overall model fit are based on F-tests.

## 4 Discussion

This study found that individual tendencies in risk and time preferences were linked to differences in physical activity and sedentary behavior. Although the associations were modest, the findings presented here add to the growing body of literature on the relationship between individual decision-making tendencies and health behaviors. The present findings suggest that greater risk aversion is associated with a tendency to spend less time sedentary, which is consistent with previous research showing that risk-averse individuals are less likely to engage in unhealthy behaviors (Anderson and Mellor, 2008; de Oliveira et al., 2016). Although no significant associations were observed between risk preferences and domain-specific physical activity, these findings suggest that risk preferences may have limited relevance to physical activity. Previous studies have similarly reported null associations, noting that risk preferences may be domain specific and that those measured in monetary contexts may not fully capture risk perceptions related to physical activity (van der Pol et al., 2017; Nicholls and Watson, 2024).

The present study found that individuals with a higher time preference seemed to be more physically active during work and less sedentary overall. This result appears to differ from those of previous studies, which reported that individuals with higher time preferences tend to be less physically active overall, including in leisure-time activities (Kosteas, 2015; Hunter et al., 2018). One possible explanation is that physical activity in the workplace may provide short-term benefits, such as completing tasks more efficiently (Grimani et al., 2019). Individuals with a higher time preference may be more responsive to immediate demands and reinforcement in structured environments like the workplace. However, previous studies have suggested that the relationship between time preference and physical activity may differ based on factors such as sex (Kosteas, 2015) and socioeconomic status (Hunter et al., 2018). Therefore, it is important to consider the possibility that the present findings reflect characteristics specific to the study population, and further research is needed in populations with different cultural and occupational backgrounds.

In this study, both risk and time preferences were associated with overall sedentary behavior. Prolonged sedentary behavior not only entails potential future health risks but may also offer limited immediate benefits for working adults, as extended sitting at work may be associated with reduced productivity (Ishii et al., 2018). These findings suggest that individual risk and time preferences may be relevant factors to consider when developing strategies to reduce sedentary behavior. Further studies are required to understand the mechanisms underlying these associations.

This study has several limitations. First, the lack of standardized measures for risk and time preferences may limit the measurement validity. In addition, using monetary-based measures may not fully capture preferences in health-related contexts. Second, cultural and environmental differences may limit generalizability. Although occupational factor was considered, residual confounding due to unmeasured aspects of the work environment cannot be ruled out. Third, self-reported questionnaires are prone to recall bias, and participants may have over- or underestimated their domain-specific physical activity and sedentary behavior. Additionally, the modest explanatory power of the models suggests that physical activity and sedentary behavior are influenced by factors not fully captured in these analyses. Finally, as the sample was drawn from an online panel, sampling bias could not be ruled out. Due to the cross-sectional design, causal relationships cannot be inferred.

## 5 Conclusion

In this study of working adults, individual risk preferences were not clearly related to domain-specific physical activity. In contrast, higher time preference was modestly associated with physical activity in work-related contexts. Given that both risk and time preferences were linked to total sedentary time, further research is needed to examine sedentary behavior across domains.

## CRediT authorship contribution statement

**Yoshino Hosokawa:** Writing – review & editing, Writing – original draft, Methodology, Formal analysis, Conceptualization, Investigation. **Kaori Ishii:** Writing – review & editing, Methodology, Formal analysis, Conceptualization, Investigation. **Ai Shibata:** Writing – review & editing, Methodology, Formal analysis, Conceptualization, Investigation. **Koichiro Oka:** Writing – review & editing, Supervision, Methodology, Formal analysis, Conceptualization, Funding acquisition, Investigation.

## Funding

This study was conducted as part of a project supported by the 10.13039/501100003478MHLW Program (Grant Number JPMH22FA1004 and JPMH25FA0101).

## Declaration of competing interest

The authors declare that they have no known competing financial interests or personal relationships that could have appeared to influence the work reported in this paper.
