# Supplementary material for: Associations of risk and time preferences with Japanese workers' physical activity and sedentary behavior
Source: Prev Med Rep. 2026 May 5;66:103491. doi: 10.1016/j.pmedr.2026.103491 (PMC13187583; doi:10.1016/j.pmedr.2026.103491)
Supplement: Supplementary material [file mmc1.docx]

**
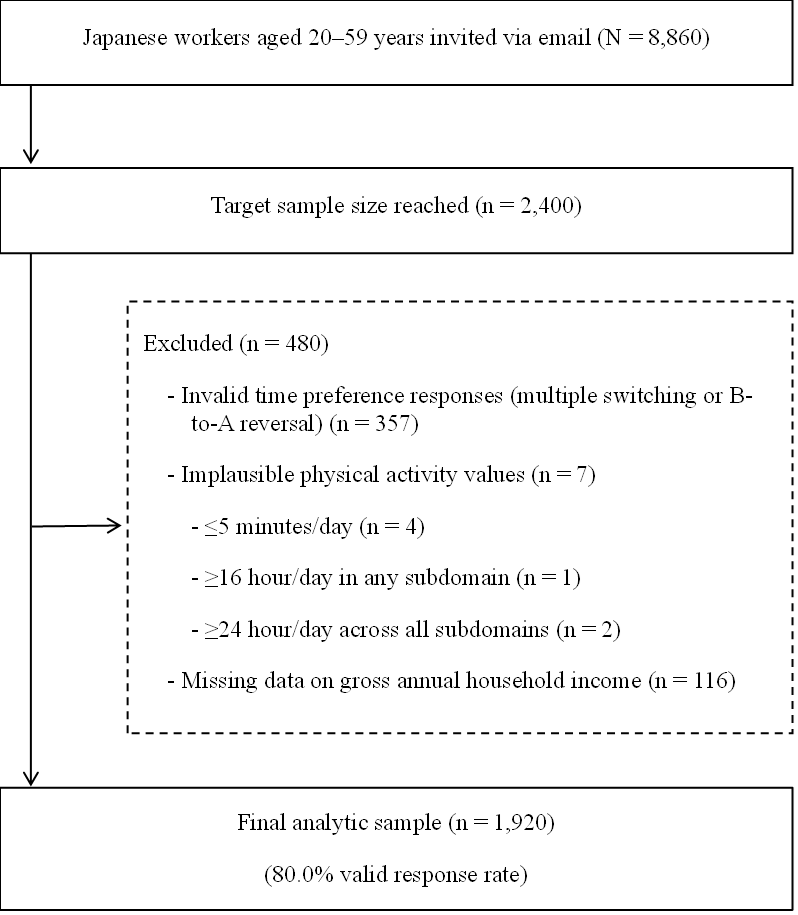
**

Supplementary Figure 1. Flow diagram of participant selection and exclusion criteria in a web-based survey of Japanese workers aged 20–59 years, March 2022.
